# Stress, anxiety, and depression in times of COVID-19: Gender, individual quarantine, pandemic duration and employment

**DOI:** 10.3389/fpubh.2022.999795

**Published:** 2022-11-03

**Authors:** Inna Levy

**Affiliations:** ^1^Department of Criminology, Ariel University, Ariel, Israel; ^2^Department of Interdisciplinary Studies, Zefat Academic College, Zefat, Israel

**Keywords:** stress, anxiety, depression, COVID-19, gender, employment, pandemic duration

## Abstract

**Objectives:**

This study explores the inter-relationship between emotional distress in adults and gender, quarantine experiences, pandemic duration, and employment.

**Methods:**

An online cross-sectional online survey comprised 943 Israelis. The link to the survey was distributed *via* different personal and academic social networking sites (e.g., Facebook, WhatsApp, and Twitter). The survey was administered using the online survey portal Google Forms. Participants addressed questions about their socio-demographic characteristics (e.g., gender, age, family status, employment, and quarantine experiences) and ranked their levels of stress, anxiety, and depression using the Hebrew version of the Depression, Anxiety and Stress Scale-DASS-21.

**Results:**

The majority of the respondents (72%) were women, 39% experienced quarantine, and 55% were unemployed. About 42% experienced a short-term pandemic (one lockdown), and the rest experienced a continuous pandemic (two lockdowns). The MANCOVA results, controlling for family status, indicated that women and unemployed participants reported higher stress, anxiety, and depression levels than men and employed participants. Participants who experienced individual quarantine reported higher anxiety and depression. Furthermore, there was a significant interaction between gender, employment, and pandemic duration. The experience of individual quarantine intensified the stress, anxiety, and depression for both employed and unemployed women. Conversely, the quarantine intensified stress, anxiety, and depression only for unemployed men, whereas the quarantine did not affect stress, anxiety, and depression among employed men.

**Conclusions:**

Employment is a critical factor regarding men's emotional state during such stressful situations as the COVID-19 pandemic. Furthermore, individual quarantine and long-term pandemics are associated with opposite outcomes regarding individual mental health. The individual quarantine is associated with increased anxiety and depression, while a long-term, continuous pandemic is associated with decreased stress.

## Introduction

With millions of people infected across 213 countries, COVID-19 has led (as of July 2022) to approximately 6.35 million deaths worldwide (1). Additionally, COVID-19 has caused an array of adverse effects on mental health, civil rights, and the economy (2–5). Many countries implemented full or partial lockdowns, with some lockdowns having lasted for lengthy periods and restricted all non-essential internal movement. People were not allowed to leave their homes even to go to work, and public places such as kindergartens and schools, parks, restaurants, and shopping malls were shut down for several months (6). The present research suggests that the emotional effects (depression, stress and anxiety) of these pandemic-related ramifications vary by gender, employment, experiences of quarantine and pandemic duration.

### COVID-19 in Israel

In Israel, COVID-19 reached pandemic status on 11 March 2020, and on 14 March 2020, the Israeli government officially imposed a strict general lockdown that lasted for a month. Israel's borders were closed during the lockdown, and the government instructed residents to remain at home while imposing limitations on the public and private sectors. Many of those who stopped working did not know when and whether they would be able to return to work (7). The second lockdown took place in mid-September 2020 and also continued for a month. The lockdowns helped to contain the pandemic but caused negative psychological effects and increased unemployment (8). In Israel, the national unemployment rate rose from 3.4 to 27% in April 2020 (7). Although both men and women were affected by the negative impacts of the economic slowdown, and the chances for unemployment were similar (9), the emotional distress related to unemployment may differ by gender.

Furthermore, as soon as the first case of COVID-19 was identified in Israel, the Israeli government established a 14-day individual quarantine (home-quarantine) rule for people who visited South Korea and Japan. By 9 March 2020, the 14-day individual quarantine was extended for all who came to Israel from abroad and those exposed to a confirmed COVID-19 patient. Therefore, besides the lockdowns, by 12 June 2022, there were about 28 million cases of individual quarantine in Israel (10).

### Quarantine experiences and gender

The research on quarantine suggests that it is unpleasant for those who experience it because it may involve separation from loved ones, financial problems, uncertainty over the situation and emotional difficulties (8, 11, 12). Recent studies that examined the effects of COVID-19 quarantine show negative psychological effects, including post-traumatic stress symptoms (PTSD), anxiety, depression, general distress, fear associated with outdoor activities, confusion and anger (5, 8, 13–16). Also, the length of quarantine is associated with an increased prevalence of PTSD and depressive symptoms, which might last after quarantine ends (14, 17).

Although these studies used the term quarantine, it is important to note that they addressed mass quarantines or general lockdowns. The individual quarantine represents a state of much stronger isolation than a lockdown (18). According to Israeli law, people in individual quarantine are not allowed to leave their homes at all (not even to buy groceries or medicine) and sometimes are not allowed to leave their rooms. Therefore, this study assumes that quarantines will be all the more so associated with the adverse psychological effects of social isolation.

H_1_: *There is a significant difference in emotional distress based on individual quarantine experience: Participants who experienced quarantine will report higher stress levels, anxiety, and depression than those who did not experience quarantine*.

As for gender differences, during COVID-19, women reported higher levels of concern, stress, anxiety and PTSD than men (19–21). Furthermore, women tend to experience worse psychological responses to lockdowns and quarantines (11, 22). These findings are consistent with prior research, showing that women demonstrate higher negative psychological responses to traumatic events than men (23, 24). One of the explanations for this pattern suggests that under extreme and unfamiliar circumstances, compared to men, women tend to feel more responsible for their children and tend to be more sensitive to the needs and feelings of those around them (25–27). Another possible explanation refers to gender socialization processes. It is socially acceptable for women to be more open about their emotions, and they are more likely than men to admit and report their fears (24, 28). Be the explanation as it may, it is possible to hypothesize that:

H_2_*: There is a significant difference in emotional distress by gender: Women will report higher stress levels, anxiety and depression than men*.

### Employment and gender during COVID-19

Employment is an important part of life as it does not just provide people with the means to make a living and improve their standard of living but also provides a sense of confidence and self-worth (29, 30). COVID-19 increased the unemployment rate and decreased hours of work and labor force participation (31). During COVID-19 lockdowns, many people lost their jobs (32) and those who were unemployed exhibited emotional and financial distress, expressed by greater symptoms of anxiety, depression and lower levels of function [(30), e.g., (33, 34)]. In general, loss of employment interrupts daily routines and harms mental well-being (35, 36).

One of the significant variables associated with the impact of unemployment is gender. Some studies found that in light of the changing patterns in earnings, unemployed married women may find unemployment just as stressful as unemployed married men (37, 38). Also, men now-a-days feel more comfortable relying on their partner's income (39). However, most studies examining the gender gap in unemployment experiences found that men experience more distress and are more negatively affected by unemployment than women [e.g., (3, 40)].

This pattern can be attributed to the differences in traditional gender roles. Men still often function as the main economic providers within the household, while many women are still more often involved in the role of caregiver and housekeeper for the household (41–43). Furthermore, as opposed to men, when women lose their job, they are more likely to replace the rewards they would gain from the job with alternative rewards from their nursing role in the household (41, 42). Therefore, in traditional cultures, a good job and financial stability may be more meaningful for men's than women's well-being. Since the Israeli culture is characterized by traditional gender norms [e.g., (44–46)], the gender gap in response to unemployment may be salient, and this study's hypothesis is:

H_3_: *There is a significant interaction between gender and employment regarding emotional distress: Unemployed men will report higher stress levels, anxiety and depression than unemployed women*.

### Pandemic duration and mental health

Considering that we have been living with COVID-19 for more than a year, it is possible to suggest that our society faces a chronic, continuous exposure to different effects of the COVID-19 pandemic. The repeated and lengthy lockdowns (6) generate unemployment (32) and increase the public's financial worries (34). Additionally, continuous pandemic exposes people to constant health-related anxiety (47). These financial and health-related worries increase people's anxiety, depression, and stress (48–50).

Although the research on effects of a prolonged pandemic is limited (51), some research has explored such concepts as chronic terrorism (52), continuous traumatic situations/stressors (53, 54), lifetime cumulative adversity (55) and ongoing traumatic stress response (56). These concepts refer to accumulating effects of continuing mass exposure to traumatic events or stressors in the context of wars and political violence such as terrorism. Inter alia, Cohen-Louck and Levy (2) suggested that the chronic state of terrorism may become predictable and controllable. Nevertheless, the research on emotional distress indicates that cumulative stress exacts a heavier toll (57), and there are severe consequences of living with ongoing traumatic stress. People who lived with continuous exposure to stress or trauma reported stronger post-traumatic distress symptoms than people who experienced a single event or short-duration traumatic exposure (57, 58). Therefore, the hypothesis is:

H_4_: *There is a significant difference in emotional distress by pandemic duration: Participants who have experienced a long-term pandemic will report higher levels of stress, anxiety and depression than participants who have experienced a short-term pandemic*.

### The current research

This study aims to determine the association between emotional distress and gender, quarantine experiences, pandemic duration, and employment. This study is unique in several ways. It focuses on the effects of individual quarantines rather than on the effects of the general lockdowns. Additionally, most COVID-19 research has been conducted during the early stages of the outbreak, whereas this study compares early and later stages. Therefore, this study allows for identifying the effects of a long-term pandemic on individual emotional well-being. Finally, by exploring the effects of employment and gender on mental health, this research may facilitate further understanding of gender-related differences and identify at-risk groups. Such information can be useful to practitioners and policymakers responsible for public mental health promotion.

## Methods

### Participants and sampling

The sample included 943 respondents. The data was collected *via* an online snowball survey. Snowball sampling is a common, non-probability method of recruiting participants. This method uses referrals from initially sampled respondents (59). The current survey was administered using the online survey portal Google Forms. In an effort to reach as many potential participants as possible, the researchers and research assistants posted the survey link on their personal and academic social networking sites (e.g., Facebook, WhatsApp, and Twitter). Also, the researchers emailed students and academic and administrative staff members from two Israeli academic institutions (the anonymized name of the university and the anonymized name of the academic college) to participate in the survey and distribute the link *via* different social networking sites.

### Measures

#### Socio-demographic characteristics

The questionnaire included questions on age, gender, marital status, educational level, and income. The time of participation was recorded automatically.

#### Depression, anxiety, and stress

This study used the Hebrew version of a short Depression, Anxiety and Stress Scale-DASS-21 to assess participants' mental health (60). High scores on the anxiety sub-scale represent a strong somatic response to fear, high scores on the depression sub-scale represent a high level of sadness and absence of incentives, and high scores on the stress sub-scale represent a higher tendency for frustration (60). Respondents scored items on a scale from 0 (did not apply to me) to 3 (apply to me very much). The scores on each subscale were computed by adding up the items and multiplying them by a factor of 2. Each subscale included seven items, and the scores for each subscale may range from 0–42. Cronbach's alpha for stress was 0.89, anxiety 0.85, and depression 0.89.

#### Pandemic duration

This study defined the individuals who participated in our survey during and shortly after the first lockdown as participants who experienced a *short-term pandemic*. The individuals who participated in the survey during and shortly after the second lockdown were considered as participants who experienced a *long-term pandemic*.

### Procedure

The Ariel University Committee of Ethics approved this study AU-SOC-KL-20200330. The instructions for the participants stated that (a) the participation in this study was anonymous, (b) participants did not have to answer questions that made them uncomfortable and could withdraw from participation at any time, and (c) the data would be used for the research purposes only. All participants gave informed consent by clicking on the “I freely consent to participate in this survey” option). There were no exclusion criteria except age, as only adults were included. The survey included three check-up questions to ensure that all participants read and understood the questions. The check-up questions directed participants to choose a specific reply. Seven participants who failed to choose the right option were excluded from the analyses. It took about half an hour to answer the questionnaire. The data was collected in two main waves: (1) following the first lockdown (1.4.2020–18.5.2020); (2) following the second lockdown (26.10.2020–17.11.2020).

### Statistical analysis

The data was analyzed using SPSS version 25. At first, the effects of demographic variables on the mental health indicators were explored using Pearson correlations, MANOVAs and ANOVAs. Then the hypothesis was examined through MANCOVA while controlling for family status. In order to include marital status as a covariant, it was re-coded into two variables: single (single = 1, all others = 0) and married (married = 1; all others = 0).

## Results

### Descriptive findings

The participants' age range was 18–89 (*Mean* = 34.39, *S.D*. = 17.23). The majority were women (71.7%), unemployed (54.85), and with a graduate or post-graduate level of education (70.3%). Sixty percent were single, 34% married, and 6% were divorced or widowers. Two-thirds (67%) reported their income lower than the average, 16.8% average, and 16.3% more than average. Also, 57.6% have experienced only one lockdown, and the rest have experienced two lockdowns.

The mean score for stress was 12.65 [*S.D*. = 11.36], the mean score for anxiety was 5.54 [*S.D*. = 7.51], and the mean score for depression was 9.56 [*S.D*. = 9.68]. Thus, participants' stress was stronger than their anxiety and depression, and depression stronger than anxiety. There was significant, weak and negative correlation between age and stress [*r*_(939)_ = −0.15, *p* < 0.001] and age and depression [*r*_(939)_ = −0.13, *p* < 0.001], but no significant correlation between age and anxiety [*r*_(939)_ = −0.06, *p* = 0.06].

MANOVA indicated a significant effect of marital status [*F*_(6, 1108)_ = 2.92, *p* < 0.01, η^2^ = 0.2] regarding measures of mental health. ANOVA showed a significant effect of marital status on stress [*F*_(2, 556)_ = 6.34, *p* < 0.01, η^2^ = 0.2], anxiety [*F*_(2, 556)_ = 6.13, *p* < 0.01, η^2^ = 0.2], and depression [*F*_(2, 556)_ = 6.64, *p* < 0.01, η^2^ = 0.3]. Participants who were single reported the highest levels of stress [*Mean* = 14.45, *S.E*. = 1.31], anxiety [*Mean* = 7.66, *S.E*. = 0.88], and depression [*Mean* = 11.57, *S.E*. = 1.16]. Divorced and widowers reported middle levels of stress [*Mean* = 10.33, *S.E*. = 2.14], anxiety [*Mean* = 6.44, *S.E*. = 1.43], and depression [*Mean* = 9.87, *S.E*. = 1.90]. While married respondents reported the lowest levels of stress [*Mean* = 8.76, *S.E*. = 0.91], anxiety [*Mean* = 4.03, *S.E*. = 0.61], and depression [*Mean* = 6.55, *S.E*. = 0.81].

According to MANCOVA, there was no significant main effect of income [*F*_(6, 1108)_ = 0.52, *p* = 0.79, η^2^ = 0.00], but a significant main effect of educational level [*F*_(3, 554)_ = 4.5, *p* < 0.01, η^2^ = 0.02]. ANOVA indicated a significant difference by education only in anxiety [*F*_(1, 556)_ = 5.22, *p* < 0.05, η^2^ = 0.01]: participants with a graduate and postgraduate level of education reported significantly lower anxiety [*Mean* = 4.68, *S.E*. = 0.68] than participants with a high-school level of education [*Mean* = 7.4, *S.E*. = 0.98]. There were no significant differences by education in stress [*F*_(2, 556)_ = 0.16, *p* = 0.69, η^2^ = 0.01] and depression [*F*_(2, 556)_ = 0.38, *p* = 0.54, η^2^ = 0.01]. Considering the weak and non-significant associations between age, income, and mental health indicators, only marital status and educational level were controlled for.

### Gender, quarantine, pandemic duration, and employment

MANCOVA (Table 1) indicated significant main effects of gender, quarantine and employment, and pandemic duration. ANCOVA indicated that women reported higher stress, anxiety and depression levels than men. Participants who experienced quarantine reported higher levels of anxiety and depression but similar stress levels compared to those who did not experience quarantine. Participants who experienced a long-term pandemic reported lower stress than those who experienced a short-term pandemic. There was no significant difference in anxiety and depression by pandemic duration. Finally, unemployed participants' stress, anxiety and depression were higher than employed participants.

**Table 1:** Results of MANCOVA and ANCOVA results regarding gender, quarantine, pandemic duration and employment.

	**Mental health during COVID-19**			**MANCOVA**	
	**Stress**	**Anxiety**	**Depression**	**3,908**	
	**Mean (S.E.)**	**Mean (S.E.)**	**Mean (S.E.)**	***F***	***η^2^***
**Gender**				12.60^***^	0.04
Female	14.10 (0.46)	6.50 (0.31)	10.49 (0.38)		
Male	9.01 (0.73)	4.47 (0.50)	7.59 (0.62)		
***F**_***ANCOVA***_*	34.29^***^	7.25^**^	16.54^***^		
***df***	1,910	1,910	1,910		
***η^2^***	0.04	0.01	0.02		
**Individual quarantine experience**				4.74^**^	0.02
No exposure	10.80 (0.52)	4.43 (0.36)	7.74 (0.44)		
Exposure	12.40 (0.68)	6.09 (0.46)	10.24 (0.58)		
***F**_***ANCOVA***_*			3.51	8.16^**^	11.73^**^
***df***	1,910	1,910	1,910		
***η^2^***	0.00	0.01	0.01		
**Pandemic duration**				2.68^*^	0.01
Short-term	12.73 (0.60)	5.62 (0.40)	9.70 (0.50)		
Long-term	10.8 (0.63)	4.90 (0.42)	8.29 (0.54)		
***F**_***ANCOVA***_*	7.24^**^	1.49	3.60		
***df***	1,910	1,910	1,910		
***η^2^***	0.01	0.00	0.00		
**Employment**				7.42^***^	0.02
Unemployed	12.92 (0.61)	6.21 (0.41)	10.72 (0.52)		
Employed	10.19 (0.61)	4.30 (0.42)	7.26 (0.52)		
***F**_***ANCOVA***_*	9.83^**^	10.55^**^	21.82^***^		
***df***	1,910	1,910	1,910		
***η^2^***	0.01	0.01	0.02		

* p < 0.05,

** p < 0.01,

*** p < 0.001.

**Figure 1:**
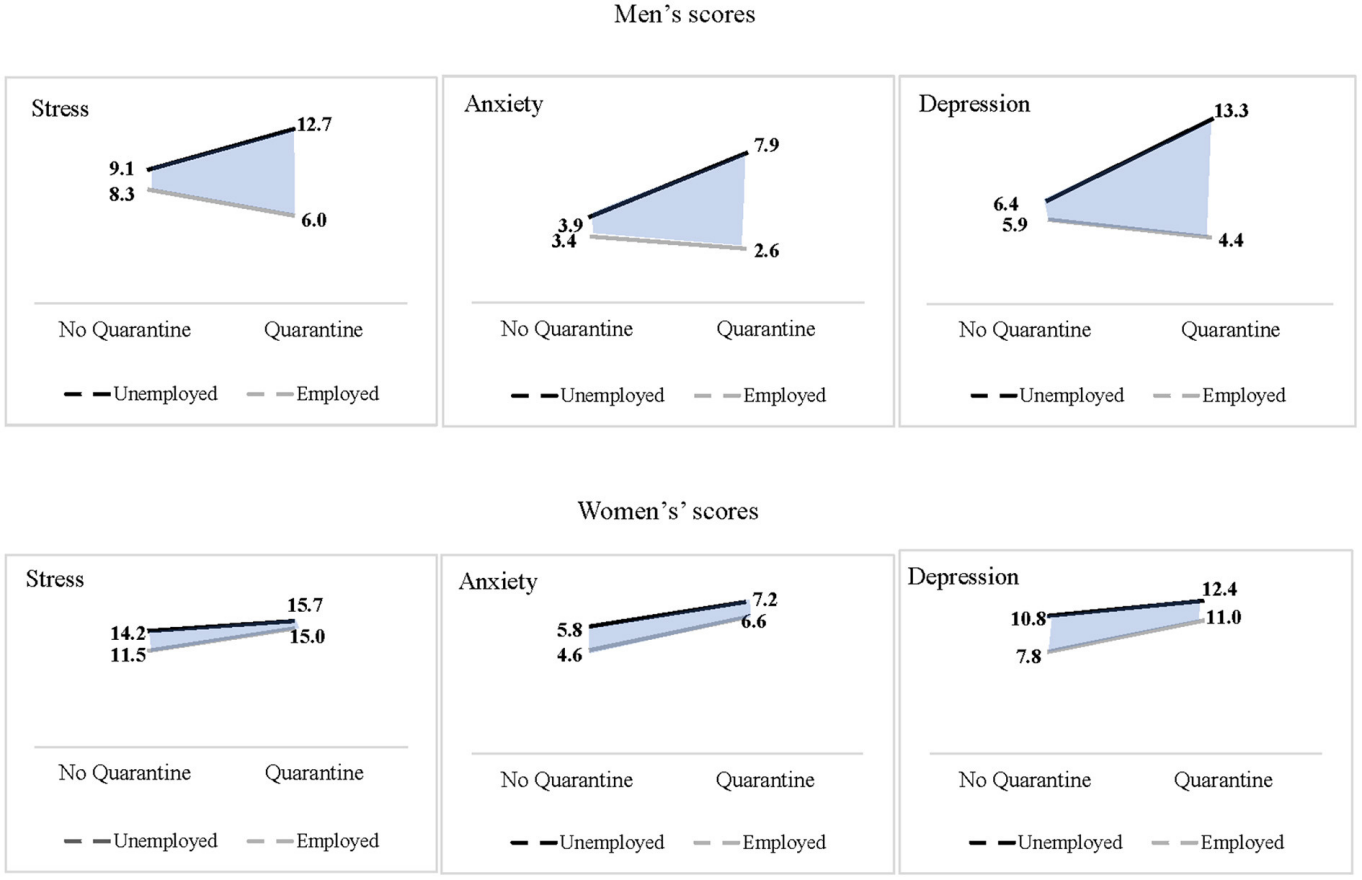
Interaction between gender, quarantine and employment regarding stress, anxiety and depression.

MANCOVA results showed no significant interaction between gender and employment [*F*_(3, 908)_ = 1.19, *p* = 0.31, η^2^ = 0.00]. However, MANCOVA indicated a significant interaction [*F*_(3, 908)_ = 3.96, *p* < 0.01, η^2^ = 0.01] between gender, quarantine and employment, and therefore several ANCOVAs were conducted. The analysis showed a significant interaction between gender, quarantine, and employment regarding stress [*F*_(1, 910)_ = 5.40, *p* < 0.05, η^2^ = 0.01], anxiety [*F*_(1, 910)_ = 5.16, *p* < 0.05, η^2^ = 0.01] and depression [*F*_(1, 910)_ = 11.70, *p* < 0.01, η^2^ = 0.01]. Figure 1 shows that among women, the effect of quarantine is additive. Women who experienced quarantine reported higher stress, anxiety, and depression, but there were no differences between employed and unemployed women. Among employed men, there was no effect of quarantine. Conversely, among unemployed men who experienced quarantine, the stress, anxiety, and depression were significantly higher than among employed men.

## Discussion

This study focuses on the interrelationship between emotional distress and gender, quarantine experiences, pandemic duration, and employment. The results showed significant differences in emotional response (stress, anxiety, and depression) based on quarantine (H_1_), employment (H_3_), and pandemic duration (H_4_). Furthermore, the present research indicates significant gender differences in emotional responses to COVID-19-related quarantine and unemployment. These findings support the notion that the COVID-19 pandemic is a stressful situation (61, 62).

A key finding of this study addresses the interrelationship between gender, unemployment and quarantine, showing that women reported higher levels of stress, anxiety and depression than men. This finding supports the hypothesis (H_2_) and prior research that women are more vulnerable than men to the adverse psychological effect of COVID-19 (19–21) and other traumatic events (23, 24). As for the interaction between gender and unemployment, contrary to the hypothesis (H_4_), the interaction was non-significant. Nevertheless, there was a significant three-way interaction between gender, unemployment and quarantine experience. Thus, both employed and unemployed women who experienced quarantine reported higher anxiety, stress, and depression levels. Conversely, the quarantine had no significant effect on employed men. However, among unemployed men, those who experienced quarantine reported higher anxiety, stress and depression than those who had not experienced quarantine.

This pattern suggests that employment may have a protective role against the adverse effects of quarantine for men, while for women, employment does not have a protective value. Due to traditional gender roles (41–43), men still value their ability to earn income and support their families (40). Traditional norms lead women to develop household chores and men to increase their workloads (46, 63), even during the COVID-19 pandemic (46). The interaction pattern also supports the assumption of precarious manhood theory (64), which states that manhood is elusive and can be lost, whereas womanhood is considered naturally based and comparatively stable and secure. Therefore, men striving to maintain their manhood must constantly affirm and publicly prove it. In traditional societies such as Israel (44–46), loss of the provider role during COVID-19 can be considered a threat to manhood. Therefore, unemployed men are more vulnerable to additional stressors such as quarantine.

Another intriguing set of findings refers to the effects of individual quarantine and pandemic duration. The results on individual quarantine impact correspond with prior studies on general lockdowns that showed a negative effect of social distancing and isolation on individual well-being [e.g., (8, 11, 12, 14, 16)]. In the current study, the participants who experienced an individual quarantine reported higher levels of anxiety and depression than those who did not experience an individual quarantine. Nevertheless, the findings only partially support our hypothesis (H_1_) since there were no significant differences in stress levels by quarantine experience. The opposite pattern emerged regarding pandemic duration. Contrary to the hypothesis (H_4_), participants who experienced a short-term pandemic reported a higher stress level than participants who experienced a long-term pandemic; there were no significant differences in anxiety and depression based on pandemic duration.

Since pandemic duration was defined by the number of lockdowns experienced (one vs. two), it can be suggested that this set of findings should be addressed in the context of individual quarantine and general lockdown characteristics. Individual quarantine represents a higher isolation state than general lockdowns (18). A lockdown aims to protect people who are “isolated” from the possibility of COVID-19 infection. A quarantine aims to protect others from the quarantined, isolated individual (who was exposed to COVID-19). Since lockdowns protect against disease transmission, people who adhere to lockdowns decrease their danger of being infected by COVID-19. In comparison, individuals who stay in quarantine have already been exposed to the virus (*via* contact with confirmed COVID-19 patients). They are in danger of and afraid of becoming sick (65). Therefore, individual quarantine might contribute to increased anxiety, less than general lockdowns. Also, lockdowns are inclusive of the general population, while quarantines are exclusive for the exposed individuals. Thus, a feeling of a shared fate during general lockdowns may protect against the possibility of depression, while the high level of isolation during quarantines may contribute to increased depression.

Furthermore, this research shows that quarantines are not associated with stress response, whereas long-term pandemic is associated with lower stress levels. Such an effect of a long-term pandemic may indicate a habituation process to the COVID-19 threat. Although this pattern contradicts the literature on ongoing, cumulative personal trauma [(57), e.g., (58)], it echoes findings from research on mass trauma (e.g., terrorism) that chronic threats are associated with lower levels of distress due to the habituation process [(52), e.g., (66, 67)]. Further studies should explore the psycho-social mechanisms that may account for the differences between the effects of individual quarantines and general lockdowns.

### Limitation and future studies

This study has some limitations. Firstly, although the sample is relatively large, the sampling was not random nor representative. The majority of the participants were women, educated, Jewish, and single. These sample characteristics may have affected the general findings, so the external validity is relatively limited. Future COVID-19 studies should examine our findings using random and representative sampling techniques. Secondly, the data is based on self-reports. Since traditional gender socialization allows women to be more open about their emotional state than men (28), the findings on gender differences may be affected by the gender gap in emotional openness. Thirdly, due to the cross-sectional nature of the survey, further research is needed to understand the causal pathways between this study's variables. Finally, perception of gender roles and manhood may vary by society's characteristics; therefore, future studies should consider exploring this study's assumption in additional cultural contexts.

## Conclusion

Employment is a significant factor regarding men's emotional state during such stressful situations as the COVID-19 pandemic. For men, the financial provider role is still meaningful and critical, despite ongoing social changes. Moreover, this study is one of the few that addressed the impact of individual quarantine and general lockdowns as represented by pandemic duration. The findings show that individual quarantine and pandemic duration are associated with different patterns of emotional distress. The individual quarantine increases anxiety and depression, while a long-term, continuous pandemic decreases stress. Although women reported higher levels of mental distress than men, this study underlines that unemployed men are especially vulnerable among individuals who have experienced quarantine.

Regarding practical implications, this study's findings suggest that government efforts to manage viral pandemics should address the employment issue. The findings also imply a need for gender-specific interventions in times of pandemic-related lockdowns and quarantines. Such programs should address women's vulnerability to the negative effects of pandemics and men's vulnerability to the combined effects of quarantines and unemployment.

## Data availability statement

The raw data supporting the conclusions of this article will be made available by the authors, without undue reservation.

## Ethics statement

The studies involving human participants were reviewed and approved by Ariel University Committee, Israel (AU-SOC-KL-20200330). The patients/participants provided their informed consent to participate in this study.

## Author contributions

The author confirms being the sole contributor of this work and has approved it for publication.

## Conflict of interest

The author declares that the research was conducted in the absence of any commercial or financial relationships that could be construed as a potential conflict of interest.

## Publisher's note

All claims expressed in this article are solely those of the authors and do not necessarily represent those of their affiliated organizations, or those of the publisher, the editors and the reviewers. Any product that may be evaluated in this article, or claim that may be made by its manufacturer, is not guaranteed or endorsed by the publisher.
